# *Mycoplasma hyopneumoniae* inhibits the unfolded protein response to prevent host macrophage apoptosis and M2 polarization

**DOI:** 10.1128/iai.00051-24

**Published:** 2024-08-12

**Authors:** Tong Liu, Yujuan Zhang, Huanjun Zhao, Qi Wu, Jiuqing Xin, Qiao Pan

**Affiliations:** 1 State Key Laboratory for Animal Disease Control and Prevention, Harbin Veterinary Research Institute, Chinese Academy of Agricultural Sciences, Harbin, China; Washington State University, Pullman, Washington, USA

**Keywords:** *Mycoplasma hyopneumoniae*, unfolded protein response, antiapoptosis, polarization, cytokines

## Abstract

Enzootic pneumonia caused by *Mycoplasma hyopneumoniae* (*M. hyopneumoniae*) has inflicted substantial economic losses on the global pig industry. The progression of *M. hyopneumoniae* induced-pneumonia is associated with lung immune cell infiltration and extensive proinflammatory cytokine secretion. Our previous study established that *M. hyopneumoniae* disrupts the host unfolded protein response (UPR), a process vital for the survival and immune function of macrophages. In this study, we demonstrated that *M. hyopneumoniae* targets the UPR- and caspase-12-mediated endoplasmic reticulum (ER)-associated classical intrinsic apoptotic pathway to interfere with host cell apoptosis signaling, thereby preserving the survival of host tracheal epithelial cells (PTECs) and alveolar macrophages (PAMs) during the early stages of infection. Even in the presence of apoptosis inducers, host cells infected with *M. hyopneumoniae* exhibited an anti-apoptotic potential. Further analyses revealed that *M. hyopneumoniae* suppresses the three UPR branches and their induced apoptosis. Interestingly, while UPR activation typically drives host macrophages toward an M2 polarization phenotype, *M. hyopneumoniae* specifically obstructs this process to maintain a proinflammatory phenotype in the host macrophages. Overall, our findings propose that *M. hyopneumoniae* inhibits the host UPR to sustain macrophage survival and a proinflammatory phenotype, which may be implicated in its pathogenesis in inducing host pneumonia.

## INTRODUCTION

Macrophages are among the first lines of defense against microbial infections; they facilitate and coordinate host immunity by secreting many cytokines (1). Polarization is crucial for macrophage biological functions. Upon different stimuli, macrophages can be polarized into two types: classically activated macrophages (M1) and alternatively activated macrophages (M2). M1 macrophages produce proinflammatory cytokines, such as TNFα, IL-6, CXCL-8, and IL-1β, which are known to have tissue-damaging, anti-infection, and antisepsis effects, and are mainly involved in the Th1-type immune response. M2 macrophages are primarily involved in the Th2-type immune response and exerts anti-inflammation, tissue repair, and immunomodulatory roles (2). The dynamic balance between M1 and M2 macrophages is critical for the organism. Although M1 macrophages can fight infections, over-polarization to M1 macrophages secretes large amounts of inflammatory cytokines/chemokines, leading to organismal immune function damage and lesions (3).

The unfolded protein response (UPR) plays a pivotal role in cellular endoplasmic reticulum (ER) homeostasis, host defense, cell survival, and innate immune signaling (4). The ER is an important eukaryotic intracellular organelle that serves as a primary site for posttranslational modification, folding, and oligomerization of synthesized proteins (5). Several factors (e.g., bacterial infection, local ischemia, and increased protein synthesis) perturb the ER homeostasis to trigger ER stress (ERS) and subsequently activates the UPR (6). The UPR contains three signaling pathways, namely, the PERK, IRE1α, and ATF6 pathway. These pathways, respectively, correspond to three sense molecules, PERK, IRE1α, and ATF6, which are activated by phosphorylation or splicing after dissociation from glucose-regulated protein 78 (GRP78, the hallmark protein of the UPR), thereby activating the downstream signaling pathways (7, 8). The UPR, on the one hand, is activated as a cytoprotective signal to mitigate ERS and restore ER homeostasis, leading to apoptosis once ER homeostasis cannot be restored (9); on the other hand, as a conserved pathway in eukaryotic cells, the UPR functions as an important signaling hub for the host. It engages in crosstalk with multiple intracellular signaling pathways and regulates each other to form a complex regulatory network in response to organismal physiological and pathological events, such as increased protein synthesis and microbial invasion (10).

The UPR is critical for macrophage survival and immune function. Immune cells are known to communicate through cytokines, which are soluble secreted proteins including interleukins, interferons (IFNs), and the tumor necrosis factor (TNF) family (11). These cytokines are synthesized, folded, and processed by ER. UPR and ER homeostasis thus plays a pivotal role in a range of physiological events associated with immunologically significant cell types, encompassing survival and function (12). Moreover, the UPR pathway acts as an immune signaling hub, orchestrating cytokine regulation at all levels in response to various physiological or pathological events within the organism, which include sensing pathogenic molecules, activating downstream signaling pathways, regulating transcription factors, and ultimately activating cytokine production (13). Therefore, the UPR is instrumental in executing immune functions such as polarization, activation, and cytokine secretion in macrophages.

*Mycoplasma hyopneumoniae* (*M. hyopneumoniae*) is one of the most economically important respiratory pathogens in swine. The infection not only diminishes the growth rate and feed conversion efficiency but also paves the way for secondary respiratory bacterial and viral infections to initiate complex swine respiratory diseases, clinically known as the porcine respiratory disease complex (PRDC) (14–16). The pathological features of *M. hyopneumoniae* infection are characterized by a prominent accumulation of mononuclear cells and infiltration of lymphocytes, plasma cells, and neutrophils in the alveolar lumina and septa. Immune cell infiltration and massive secretion of different proinflammatory cytokines have been proven to be associated with the occurrence of *M. hyopneumoniae*-induced pneumonia in swine (17). However, the mechanism by which *M. hyopneumoniae* causes host immune cells to secrete large amounts of inflammatory cytokines is unclear. Our previous study demonstrated that *M. hyopneumoniae* specifically inhibits porcine alveolar macrophage (PAM) UPR, a process that plays a key role in PAM survival and immune function (18). We hypothesized that the *M. hyopneumoniae*-induced UPR inhibition might be linked to the sustained secretion of inflammatory cytokines in PAMs. In this study, we elucidated that *M. hyopneumoniae* inhibits PAM UPR and its three branches from undergoing apoptosis to maintain survival. It also prevents them from polarizing into the M2 macrophage phenotype, which in turn maintains their proinflammatory M1 phenotype to secrete large amounts of inflammatory cytokines. Our results suggest that *M. hyopneumoniae* inhibits PAM UPR to maintain survival and a proinflammatory phenotype, which may be associated with its causing host immune damage.

## RESULTS

### *M. hyopneumoniae* inhibits PTEC and PAM apoptosis

**Fig 1:**
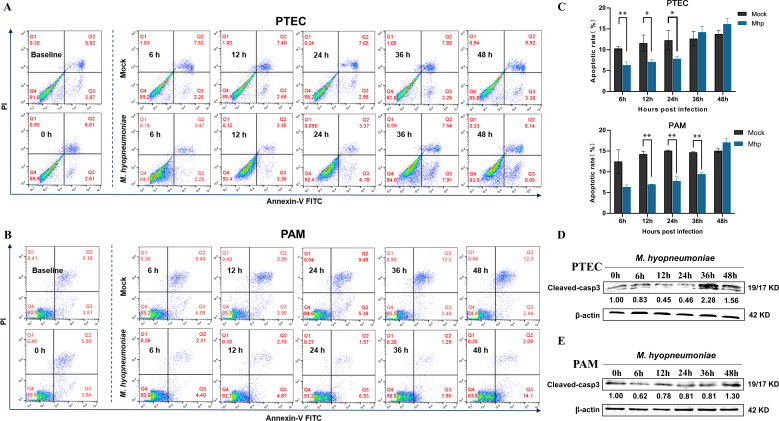
*M. hyopneumoniae* inhibits PTEC and PAM apoptosis. PTECs (3.2 × 10^5^ cells/well) and PAMs (6.0 × 10^5^ cells/well) were separately plated in twelve-well plates, incubated with or without *M. hyopneumoniae* (1.0 × 10^7^ CFU/mL) for 6, 12, 24, 36, and 48 hours. The samples were collected to analyze the apoptotic rate of PTECs (**A**) and PAMs (**B**) by flow cytometry using annexin-V-FITC-PI staining. Apoptotic rates before seeding into cell plates (baseline values) and before inoculation with *M. hyopneumoniae* (0 h) are shown on the left of panels A and B. The apoptotic rates of PTECs and PAMs infected or mock-infected with *M. hyopneumoniae* are presented in bar graphs (**C**) ,or the samples were collected to detect the protein levels of cleaved-caspase 3 activation form (cleaved-casp3) and β-actin by Western blotting. The protein levels were quantified by ImageJ and normalized to β-actin (**D and E**). The data are presented as the means ± the SDs from three independent experiments, and the significance was assessed by a two-tailed Student’s *t-test* relative to the mock cells. *, *P  <* 0.05; **, *P  <* 0.01.

Survival is a prerequisite for cells to perform normal physiological functions. We first investigated the cell survival of primary porcine tracheal epithelial cells (PTECs) and alveolar macrophages (PAMs) following *M. hyopneumoniae* infection. Annexin-V-FITC and PI staining were applied to analyze the apoptotic percentage in *M. hyopneumoniae*-infected PTECs and PAMs compared to analysis of mock-infected cells by FACS flow cytometry. The assay allowed viable (negative for both annexin-V FITC and PI staining, Q1-LL), necrotic (negative for annexin-V FITC and positive for PI, Q1-UL), early apoptotic (positive for annexin-V FITC and negative for PI, Q1-LR), and late apoptotic cells (positive for both annexin-V FITC and PI, Q1-UR) to be quantified. The results showed that the apoptotic levels (early apoptosis Q1-LR + late apoptosis Q1-UR) were significantly reduced within 24 hours in PTECs and 36 hours in PAMs after infection with *M. hyopneumoniae* compared to the mock-infection cells (Fig. 1A through C). Apoptosis is executed by caspases, a family of cysteine-dependent aspartate-directed proteases that cleave specific target proteins (19). Caspase-3 is the key terminal caspase, and its cleavage activation is an important apoptotic marker (20, 21). We detected caspase-3 cleavage levels in *M. hyopneumoniae-*infected PTECs and PAMs and found that *M. hyopneumoniae* decreased caspase-3 cleavage activation, consistent with the flow cytometry results (Fig. 1D and E). Altogether, these findings indicate that *M. hyopneumoniae* inhibits PTEC and PAM apoptosis in the early infection stage.

### *M. hyopneumoniae* impedes STS-induced host cell apoptosis

**Fig 2:**
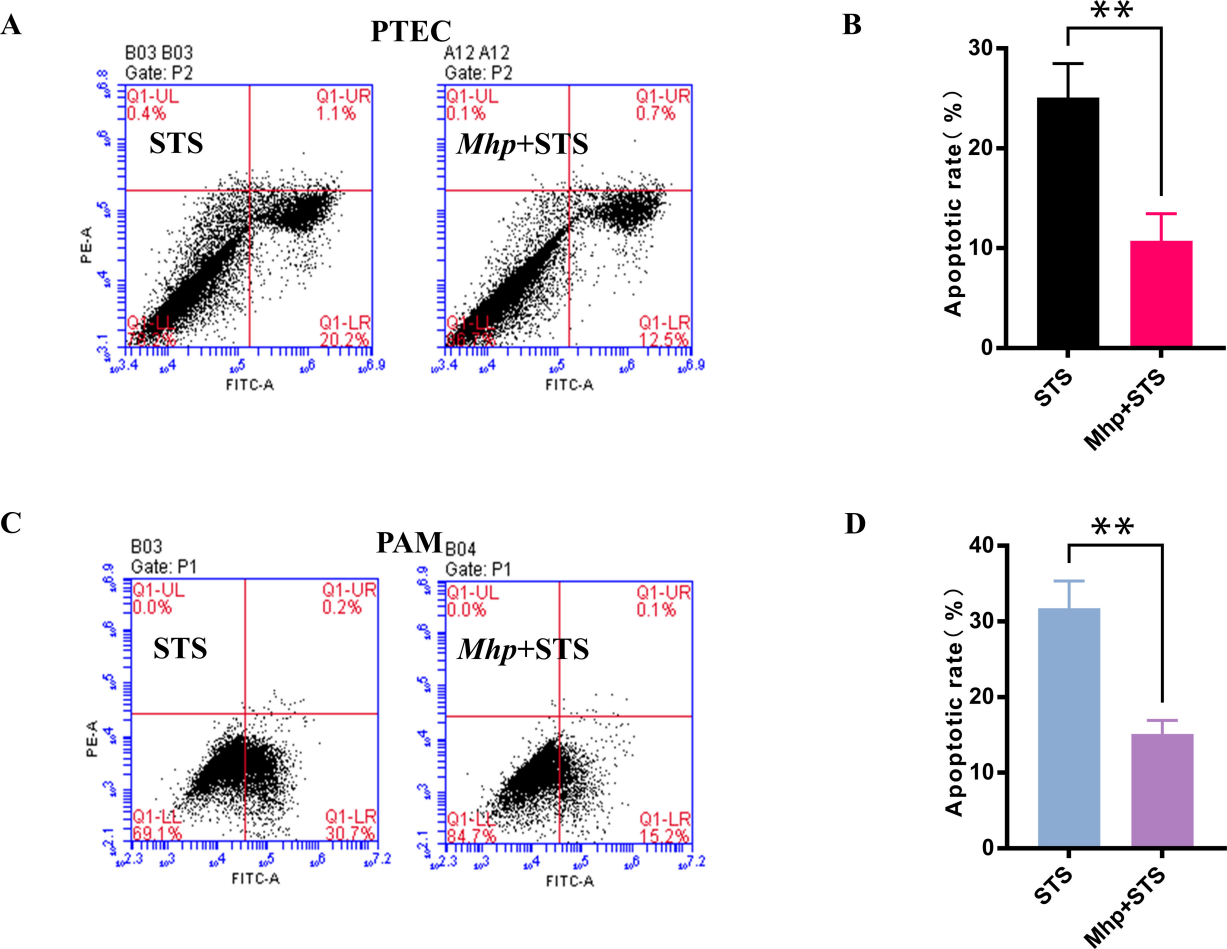
*M. hyopneumoniae* impedes STS-induced apoptosis. PTECs (3.2 × 10^5^ cells/mL) and PAMs (6.0 × 10^5^ cells/mL) were incubated with PBS or *M. hyopneumoniae* (1.0 × 10^7^ CFU/mL) for 18 hours, followed by the addition of staurosporine (STS) and further incubation for 6 hours, and the apoptotic percentage of PTECs (**A and B**) and PAMs (**C and D**) was examined by flow cytometry using annexin-V-FITC-PI staining. All data are presented as the means ± SDs from three independent experiments, and significance was assessed by a two-tailed Student’s t test. **, *P  <* 0.01.

Staurosporine (STS) is a routine apoptotic inducer that has been proven effective in inducing the apoptosis of cultured mammalian cells. We observed that the addition of STS was able to induce apoptosis in PTECs at a rate of 25.1%. Interestingly, when PTEC cells were preincubated with *M. hyopneumoniae* and then STS was added, the percentage of PTECs undergoing STS-driven apoptosis was significantly reduced to an average of 10.7% (Fig. 2A and B). Furthermore, we examined the apoptotic rate in the same treated PAM cells as described previously and found that preincubation with *M. hyopneumoniae* significantly decreased the STS-driven apoptosis rate of 31.7% to 15.1% (Fig. 2C and D). These results suggest that *M. hyopneumoniae* prevents PTECs and PAMs from undergoing apoptosis.

### *M. hyopneumoniae* LAMPs induce host cell apoptosis

**Fig 3:**
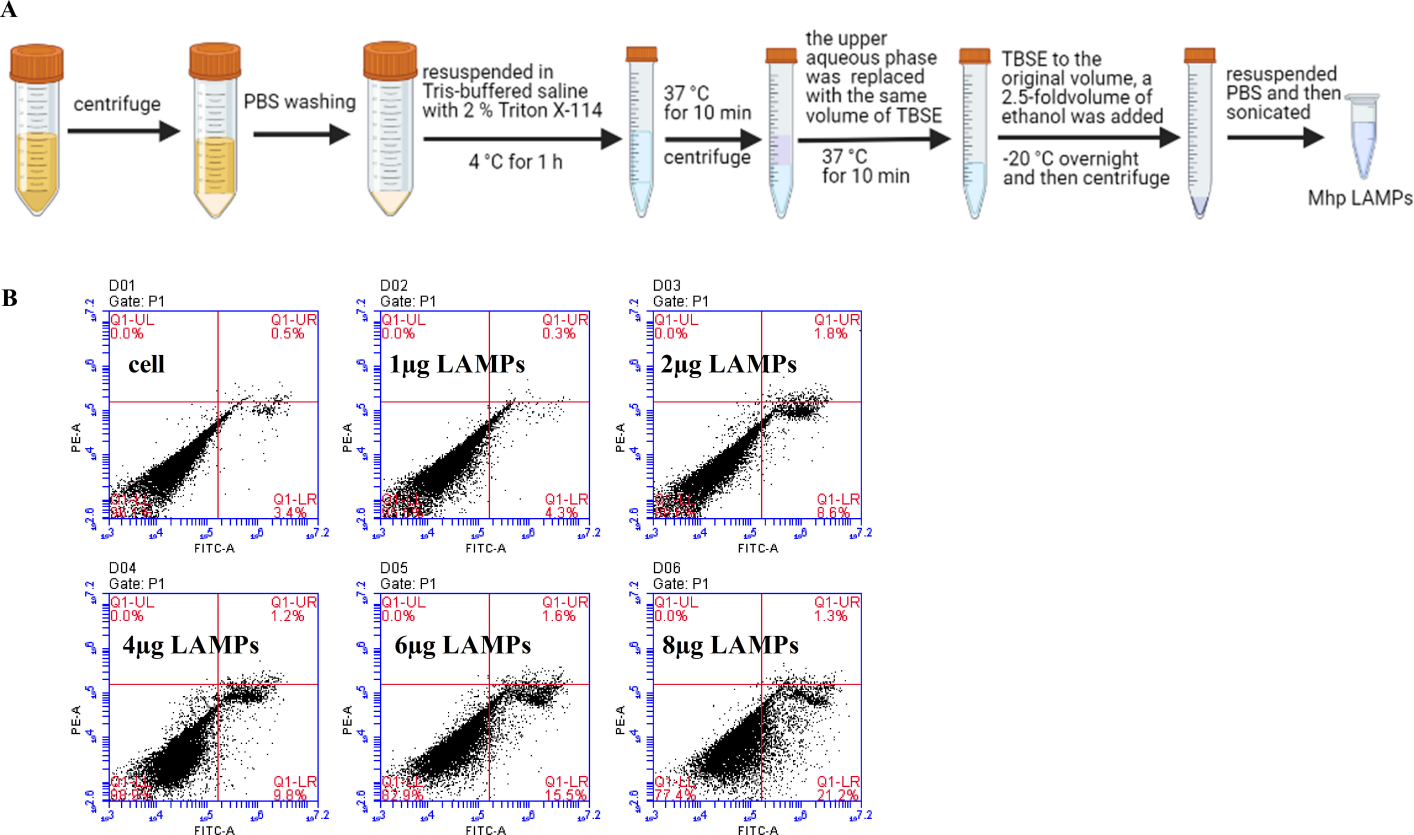
*M. hyopneumoniae* LAMPs induce host cell apoptosis. (**A**) The pipeline of *M. hyopneumoniae* LAMP extraction. (**B**) PTECs were stimulated by *M. hyopneumoniae* LAMPs (0, 1, 2, 4, 6, and 8 µg/mL) for 12 hours and then collected to assay the apoptotic level by flow cytometry using Annexin-V-FITC-PI staining.

Interactions of the *M. hyopneumoniae* lipid-associated membrane proteins (LAMPs) with the host cells are one of the major factors in mycoplasma pathogenesis (22). We extracted *M. hyopneumoniae* LAMPs and subsequently incubated PTECs with different concentrations of LAMPs for 12 hours (Fig. 3A and B). Annexin-V-FITC and PI staining were applied showing that the apoptotic rates were increased in a LAMP concentration-dependent manner, compared to control cells untreated with LAMPs. These data suggest that selected concentrations of *M. hyopneumoniae* membrane protein extracts actively induce host cell apoptosis.

### *M. hyopneumoniae* inhibits host cell apoptosis through the UPR- and caspase-12-mediated ER-associated intrinsic apoptotic pathway

**Fig 4:**
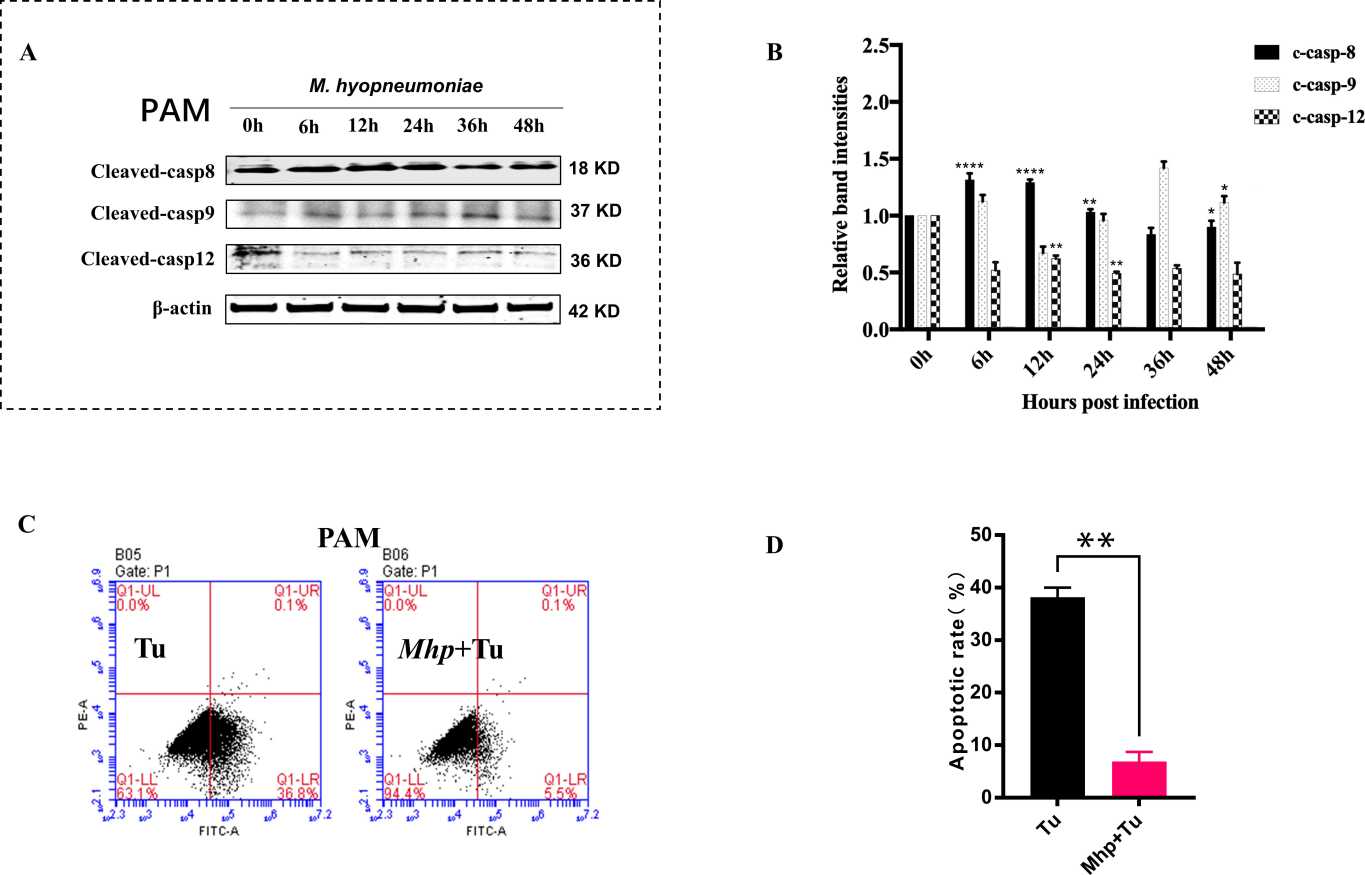
*M. hyopneumoniae* inhibits host cell apoptosis through ER-associated apoptotic signaling. (**A and B**) PAMs (6.0 × 10^5^ cells/mL) were infected by *M. hyopneumoniae* (1.0 × 10^7^ CFU/mL) for 0, 6, 12, 24, 36, and 48 hours. The protein levels of cleaved-casp8, -casp9, and -casp12 were measured by Western blotting. The data were normalized to the corresponding values in control (0 h). (**C and D**) PAMs (6.0 × 10^5^ cells/mL) were incubated with PBS or *M. hyopneumoniae* (1.0 × 10^7^ CFU/mL) for 18 hours, followed by the addition of tunicamycin (Tu) and further incubation for 6 hours, and the apoptotic rates of PAMs were checked by flow cytometry using Annexin-V-FITC-PI staining. All assays were performed with three independent experiments, and values are represented as means ± SDs. Significance was assessed by one-way ANOVA with Dunnett’s multiple comparison test, relative to the control (0 h) (**B**) or by two-tailed Student’s *t-test* (**D**). *, *P  <* 0.05; **, *P  <* 0.01; ****, *P  <* 0.0001.

Apoptosis is typically triggered by extrinsic and/or intrinsic apoptotic pathways (23). Each apoptotic pathway activates its own initiator caspase, the extrinsic (death receptor-mediated) and/or intrinsic (mitochondrial- and endoplasmic reticulum (ER)-mediated) apoptotic pathways, which are initiated separately by caspase-8,-9, and -12, which in turn activates the executioner caspase-3, resulting in nuclear and cytosolic morphological changes and finally in cell death (24). To elucidate the pathway by which *M. hyopneumoniae* prevents host cell apoptosis, Western blot assays were performed and revealed an increased in both caspase-8 and -9 cleavage activation and a reduction in caspase-12 cleavage activation, indicating that *M. hyopneumoniae* likely inhibits host cell apoptosis by interfering with the ER-related intrinsic apoptotic pathway (Fig. 4A and B).

The unfolded protein response (UPR), in addition to caspase-12, was discovered to trigger ER-mediated apoptosis (25). We next detected how *M. hyopneumoniae* affected the host UPR-induced apoptosis to determine whether it suppressed the ER-mediated intrinsic apoptotic pathway. Tunicamycin (Tu) is a common UPR activator, which was added in PAMs to induce an apoptosis rate of 38.2%. However, the apoptotic rate in PAMs with Tu added after preincubation with *M. hyopneumoniae* was only 6.9%, indicating that *M. hyopneumoniae* also inhibits UPR-triggered apoptosis (Fig. 4C and D).

Collectively, *M. hyopneumoniae* inhibits host cell apoptosis through ER-associated intrinsic apoptotic signaling (caspase-12- and UPR-mediated).

### *M. hyopneumoniae* disrupts three UPR branches to inhibit host UPR

**Fig 5:**
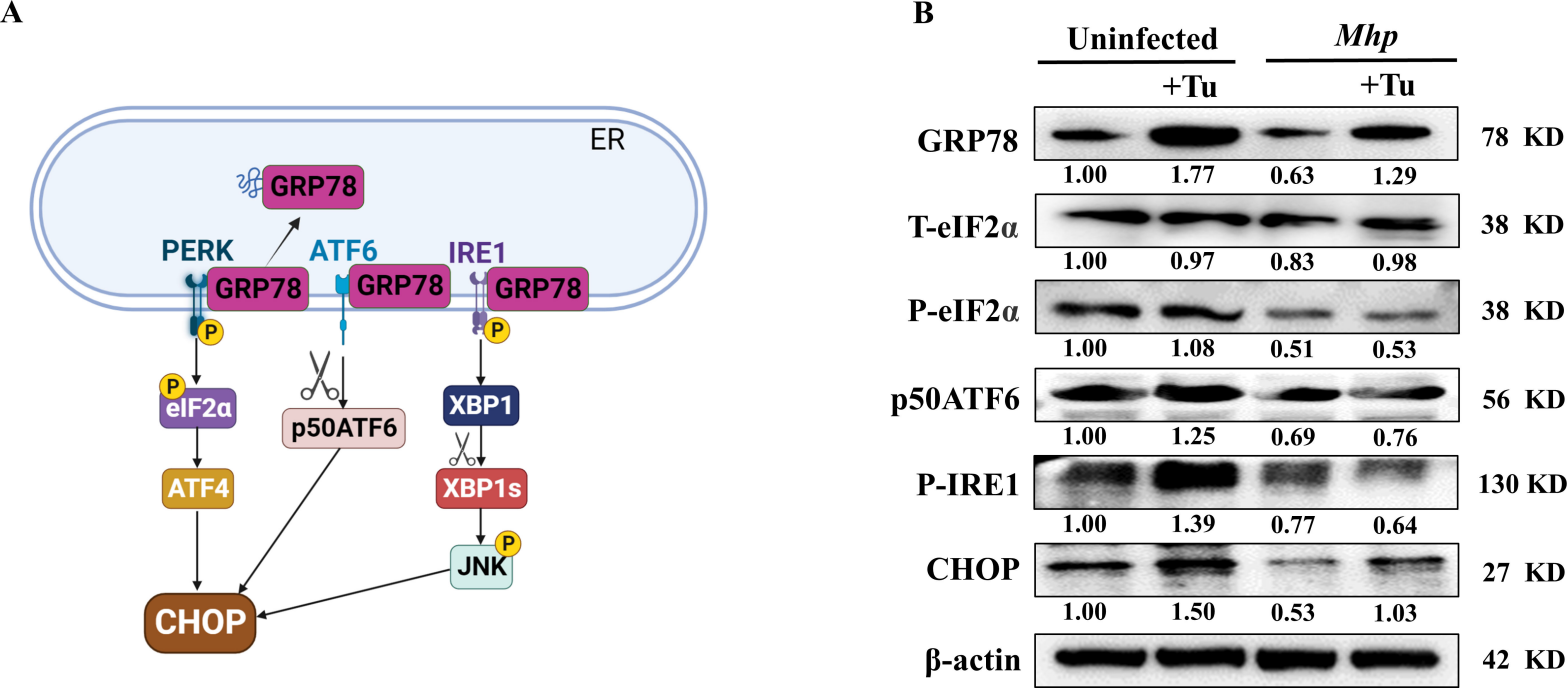
*M. hyopneumoniae* disrupts three UPR branches. (**A**) Schematic showing three UPR branches in the ER. (**B**) PAMs were incubated with *M. hyopneumoniae* or an equal volume of PBS for 18 hours, followed by the addition of Tu (2  µg/mL) or DMSO and further incubation for 6 hours. The protein levels of GRP78, total-eIF2α (T-eIF2α), P-eIF2α (phospho-eIF2α), p50ATF6 (ATF6 cleavage-activation form), phosphor-IRE1 (P-IRE1), CHOP, and β-actin were quantified by Western blotting. The protein levels were quantified by ImageJ and normalized to β-actin.

GRP78 and CHOP are hallmark molecules for UPR activation and UPR-induced apoptosis, respectively (Fig. 5A). We utilized PBS or *M. hyopneumoniae* to incubate PAM cells with or without the subsequent addition of Tu and then assayed the expression of these two molecules. *M. hyopneumoniae* was observed to inhibit the expression of both molecules compared to the uninfected group. Notably, Tu, a conventionally used UPR inducer, significantly upregulated the GRP78 and CHOP expression. Interestingly, Tu-induced upregulation of these two molecules in PAMs was alleviated or counteracted by pretreatment with *M. hyopneumoniae*, reaffirming that *M. hyopneumoniae* inhibits the host UPR and its mediated ER-associated intrinsic apoptotic signaling (Fig. 5B).

UPR consists of three signaling pathways: protein kinase R-like endoplasmic reticulum kinase (PERK), activating transcription factor 6 (ATF6), and inositol-requiring enzyme 1 (IRE1) (26) (Fig. 5A). To determine which signaling pathway *M. hyopneumoniae* employs to inhibit the UPR in order to block the ER-mediated apoptotic pathway, we treated PAMs as described previously and tested the key molecule activation on the three UPR pathways. The results showed that *M. hyopneumoniae* repressed all the three UPR pathways, as evidenced by the reduced level of phosphorylation activation of eukaryotic translation initiation factor 2 alpha (eIF2α) and IRE1, as well as the level of ATF6 cleavage activation. *M. hyopneumoniae* even actively attenuates and blocks the Tu activation on these three UPR signaling pathways (Fig. 5B).

Taken together, these data suggest that *M. hyopneumoniae* infection suppresses all the three UPR branches to suppress the host UPR.

### *M. hyopneumoniae* blocks UPR activation-triggered M2 polarization of PAMs

**Fig 6:**
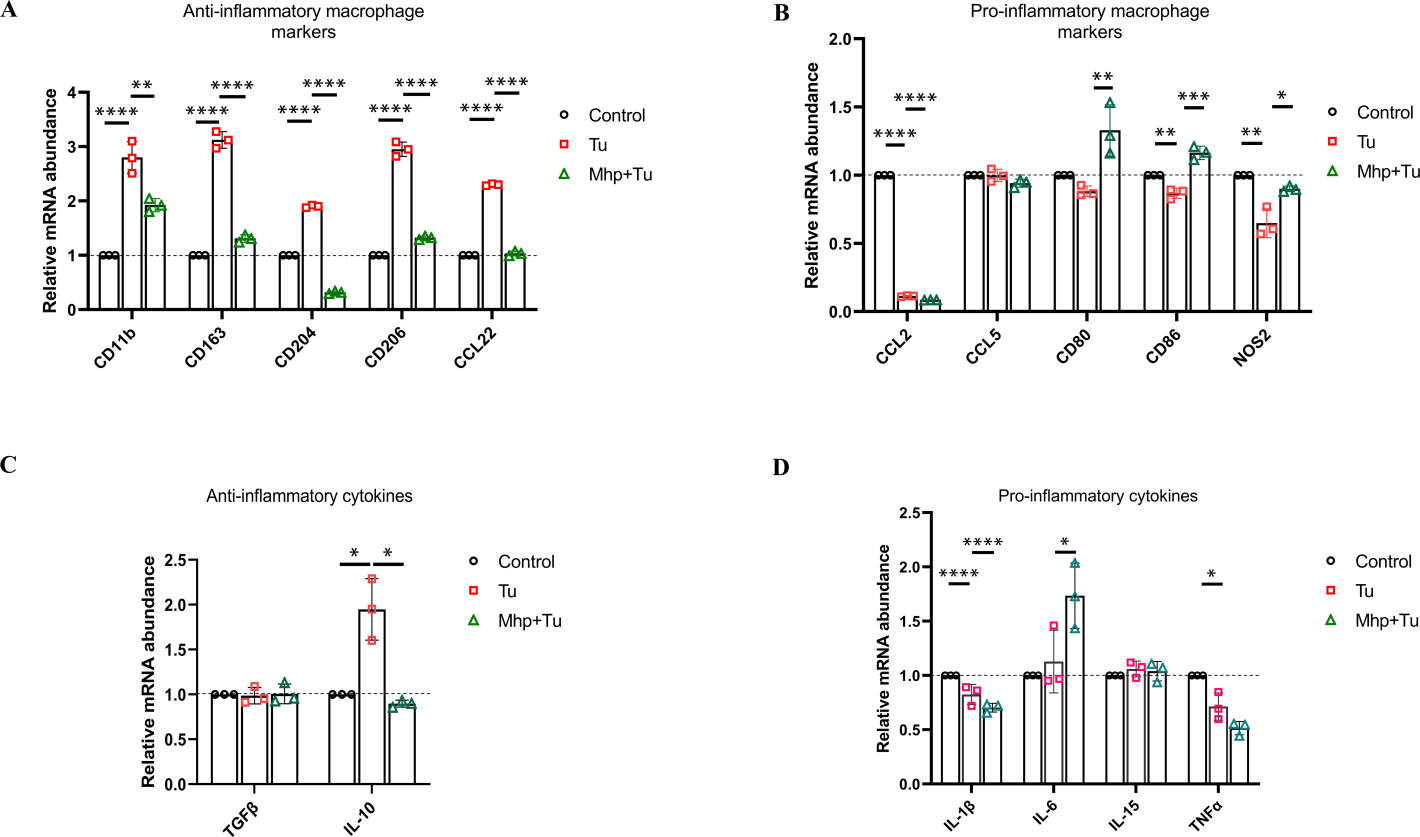
*M. hyopneumoniae* blocks UPR activation-triggered M2 polarization of PAMs. Expression of macrophage anti-inflammatory markers (**A**), pro-inflammatory markers (**B**), anti-inflammatory cytokines, (**C**) and proinflammatory cytokines (**D**) in PAMs. PAMs were incubated with *M. hyopneumoniae* (1.0 × 10^7^ CFU/mL) or an equal volume of PBS for 18 hours, followed by the addition of Tu (2  µg/mL) and further incubation for 6 hours. Cells were collected for qPCR detection. The data are presented as the means ± SDs from three independent experiments, and significance was assessed by one-way ANOVA with Tukey’s multiple comparison test. *, *P  *< 0.05; **, *P  <* 0.01; ***, *P  <* 0.001; ****, *P  <* 0.0001.

To elucidate the impact of *M. hyopneumoniae* blocking UPR activation on macrophage polarization, we analyzed the expression of pro- and anti-inflammatory markers in PAMs exposed to Tu or incubated with *M. hyopneumoniae* before exposure to Tu. Interestingly, exposure to Tu led to further increase in expression of many anti-inflammatory markers, such as surface receptors CD11b, CD163, CD204, CD206, and chemokine CCL22 (Fig. 6A), while causing a trend for decreased expression of distinct markers of inflammation, such as CCL2, CD86, and NOS2 (Fig. 6B). The Tu-treated PAMs exhibit an anti-inflammatory phenotype (M2 polarization). However, preincubation of *M. hyopneumoniae* blocks Tu-triggered M2 polarization of PAMs, as evidenced by the fact that *M. hyopneumoniae* actively prevented the upregulation of these anti-inflammatory markers and downregulation of these proinflammatory markers by Tu (Fig. 6A and B). Analysis of cytokine expression showed a similar result: anti-inflammatory cytokine IL-10 expression was significantly upregulated by Tu, whereas it did not show an increase after pretreatment with *M. hyopneumoniae* (Fig. 6C). *M. hyopneumoniae* significantly promotes the release of the proinflammatory cytokine IL-6 (Fig. 6D).

In summary, *M. hyopneumoniae* prevents UPR activation and its induced shift of PAMs toward an anti-inflammatory phenotype.

## DISCUSSION

Mycoplasma regulation on host apoptosis has been explored in several studies. Most of the studies were engaged with the direct effect of Mycoplasma or its components on the apoptotic processes in infected cells. It has been shown that *M. ovipneumoniae* triggers sheep airway epithelial cell apoptosis, and *M. genitalium* lipoproteins induce human monocytic cell apoptosis (27, 28). In contrast, there are also several studies indicating that mycoplasma infections do not induce apoptosis or even prevent apoptosis. *M. fermentans* was observed to inhibit TNF-α-induced apoptosis in the human myelomonocytic U937 cell line (29). Similarly, the infection of *M. bovis* delayed apoptosis in bovine monocytes (30). Indeed, bacteria (31), viruses (32), and parasites (33) can either induce or prevent apoptosis to augment infection. The differential regulation on host apoptosis of mycoplasmas may be attributed to their strain characteristics, live mycoplasmas or components, infection stage, and host cell types.

To our knowledge, this is the first report presenting the ability of *M. hyopneumoniae* to inhibit host apoptosis. In this study, we used primary porcine tracheal epithelial cells (PTECs) and alveolar macrophages (PAMs), which are susceptible to *M. hyopneumoniae*, to characterize the infected cell apoptosis. Morphological and biochemical features of apoptosis, such as membrane-bound protein V staining and apoptotic terminal caspase activation, were evaluated in *M. hyopneumoniae*-infected cells. The findings revealed that the apoptosis was not induced by *M. hyopneumoniae* in PTECs within 36 hours and PAMs within 48 hours. Notably, *M. hyopneumoniae* significantly reduced PTEC and PAM apoptosis, even in the presence of the apoptosis inducer STS. Furthermore, we tested the apoptosis inhibitory action of membrane proteins (LAMPs), the main antigenic component of *M. hyopneumoniae*, but did not observe this feature. We hypothesize that this may be related to the inappropriate concentrations we chose or to the fact that live *M. hyopneumoniae* is necessary for inhibition of host cell apoptosis.

Apoptosis is typically triggered by extrinsic (death receptor-mediated) and/or intrinsic (mitochondrial- and endoplasmic reticulum (ER)-mediated) apoptotic pathways, which are separately initiated by caspase-8,-9, and 12 and subsequently converge on the downstream executioner caspase-3 (25, 26). Caspases, a family of cysteine-dependent aspartate-directed proteases, play a central role in the initiation and execution of apoptosis by cleaving a large number of proteins (20). Several studies have demonstrated that infections frequently target the intrinsic apoptotic pathway to avoid or counteract host cell apoptosis. *Anaplasma phagocytophilum*, for example, secretes Ats-1 proteins that target host mitochondria to prevent mitochondria-mediated intrinsic apoptotic pathway activation (34, 35), and *Brucella abortus* VceC suppresses the ER-mediated intrinsic apoptotic pathway in goat trophoblast cells (36).

To elucidate the apoptotic pathways adopted by *M. hyopneumoniae* to inhibit host cell apoptosis, we examined the activation of key caspases on each classical apoptotic pathway. The data indicated that *M. hyopneumoniae* predominantly impeded caspase-12 activation, namely, the ER-associated intrinsic apoptotic pathway. As already known, in addition to caspase-12, the unfolded protein response (UPR) is also able to activate ER-associated intrinsic apoptosis (37). We then examined the impact of *M. hyopneumoniae* on UPR-triggered ER-associated apoptosis and found that *M. hyopneumoniae* positively blocked the induction of PAM cell apoptosis by the UPR activator Tu. Hence, *M. hyopneumoniae* selectively targeted ER-associated intrinsic apoptosis (both caspase-12- and UPR-mediated) to in turn suppress host cell apoptosis. Our further results also showed that *M. hyopneumoniae* robustly inhibits the three UPR branches and their induced apoptosis.

*M. hyopneumoniae* specifically inhibited PAMs UPR, likely affecting PAM immune function, given that the UPR is essential for macrophage survival and immune function performance (38). Polarization is the process by which macrophages differentiate into proinflammatory or anti-inflammatory phenotypes to exhibit different immune functions (39). M1 macrophages mainly secrete proinflammatory cytokines to involve in immune responses such as anti-infection and anti-tumor, whereas M2 macrophages primarily secrete anti-inflammatory cytokines to engage in anti-inflammatory and tissue repair immune processes (40). We observed that UPR activation significantly upregulated the expression of anti-inflammatory markers and cytokines in PAMs, aggressively driving PAM M2 polarization. Intriguingly, *M. hyopneumoniae* obstructed this action induced by UPR activation, preventing PAM M2 polarization. It appears to be for the purpose of maintaining its M1 type (proinflammatory phenotype). This result is consistent with *M. hyopneumoniae* infection in pigs causing massive macrophage infiltration and cytokine release.

**Fig 7:**
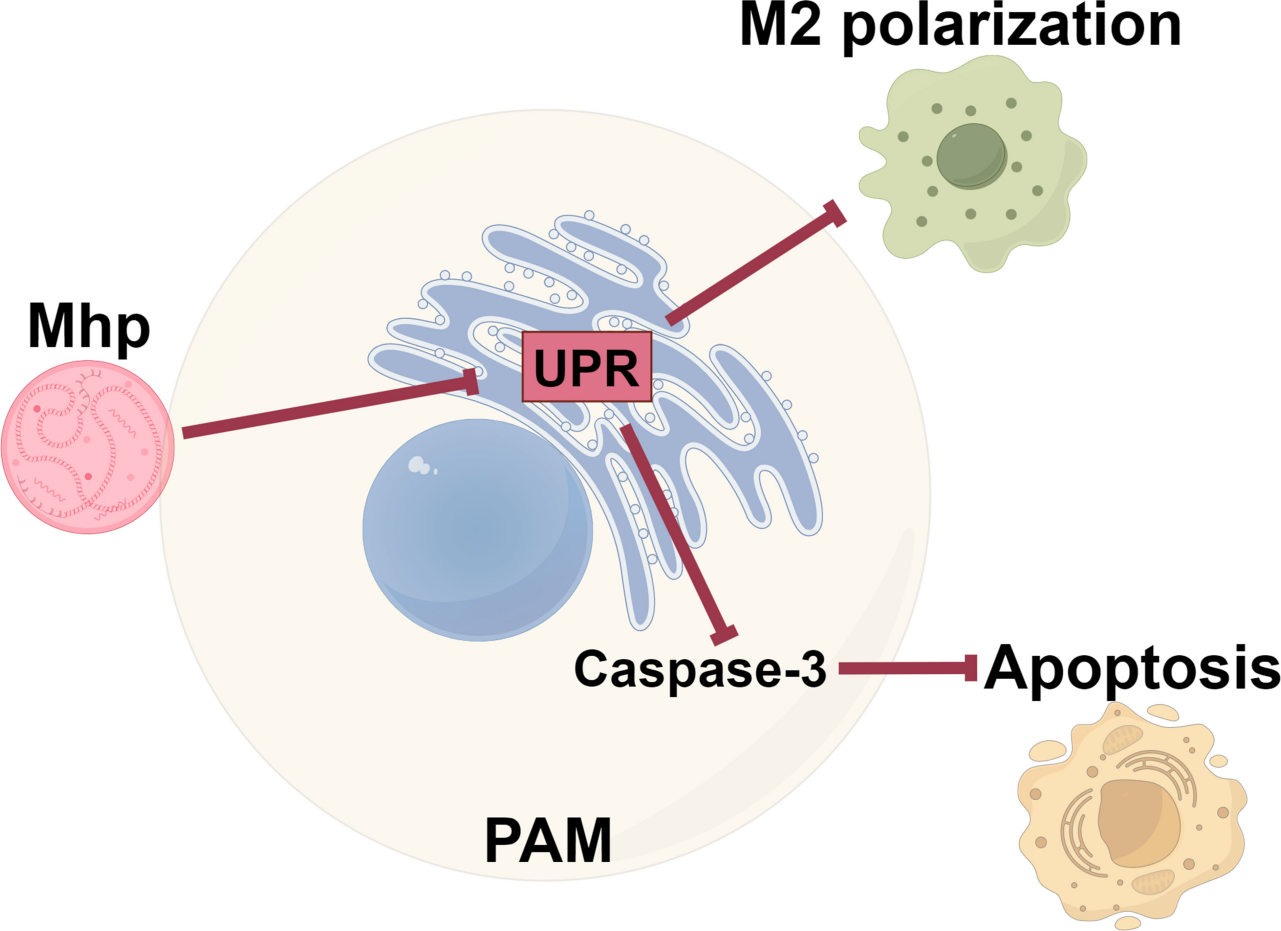
Model depicting *M. hyopneumoniae* inhibition on the UPR to prevent host macrophage apoptosis and M2 polarization (the figure was drawn by Figdraw).

In conclusion, we report here that *M. hyopneumoniae* confers resistance to apoptosis and M2 polarization on host macrophages by inhibiting the UPR (Fig. 7), thereby maintaining macrophage survival and a proinflammatory phenotype. This mechanism likely represents an important countermeasure exhibited by *M. hyopneumoniae* to avert succumbing to the cell death machinery and may be involved in pig mycoplasma pneumonia pathogenesis.

## MATERIALS AND METHODS

### Mycoplasma strains, cells, and culture conditions

*M. hyopneumoniae* (ATCC25095) were purchased from American Type Culture Collection (ATCC) and cultivated in a mycoplasma medium (Basal Media, Shanghai, China) at 37°C. To estimate the numbers of CFU in the cultures, serial dilutions were plated on a modified pleuropneumonia-like organism (PPLO) medium containing 1.5% agarose (V2111; Promega) and incubated at 37°C. The CFU was counted 7–10 days later using a microscope (41). *M. hyopneumoniae* was pelleted by centrifugation at 10,000 g for 10 minutes and resuspended to the designated concentration CFU/mL in phosphate-buffered saline (PBS).

Primary porcine tracheal epithelial cells (PTECs) and porcine alveolar macrophages (PAMs) were separately prepared from the tracheas and lungs of two 5-week-old SPF piglets using previously described protocols (42). PTECs and PAMs were cultured in Dulbecco’s modified Eagle medium (DMEM) (Gibco) supplemented with 10% heat-inactivated fetal bovine serum (Gibco) and 1% antibiotic–antimycotic solution (Gibco), and 10 mM HEPES (Invitrogen), and incubated at 37°C in 5% CO_2_. PTECs were cultivated for 4 days per generation and used at low passage number (< 5). Since PAMs cannot be passaged *in vitro*, we collected PAMs and then seeded them into the cell plates for subsequent experiments.

### *Mycoplasma hyopneumoniae* infection and treatments with chemicals

PTECs (3.2 × 10^5^ cells/mL) and PAMs (6.0 × 10^5^ cells/mL) were plated in twelve-well plates. Upon attaining 90% cell confluence, PTECs or PAMs were infected with *M. hyopneumoniae* (1.0 × 10^7^ CFU/mL) and then treated with different concentrations of chemicals or the same volume of DMSO. After incubation for 24 hours, cells were harvested and then subjected to Western blot and apoptosis assay.

Staurosporine (STS) and tunicamycin (Tu) were purchased from Sigma-Aldrich. These chemicals were dissolved in dimethyl sulfoxide (DMSO).

### Apoptosis assay

PTECs (3.2 × 10^5^ cells/mL) and PAMs (6.0 × 10^5^ cells/mL) were plated in twelve-well plates. Upon attaining 90% cell confluence, the medium was replaced with DMEM supplemented with 1% antibiotic–antimycotic solution and 10 mM HEPES. The cells were further incubated for 6 hours, the medium was removed, the cells were washed with PBS (pH 7.4), and incubated with *M. hyopneumoniae* (1.0 × 10^7^ CFU/mL). Non-treated cells were used as controls. After incubation for different hours, the cells were harvested by trypsinization. After being washed with PBS twice, the cells were stained with Annexin-V-FITC (BD Biosciences) and propidium iodide (PI, BD Biosciences) in the dark for 15 minutes at room temperature. Finally, 400 µL 1 × binding buffer was added to each tube. Apoptotic rates of the cells were assessed using a flow cytometry system (Accuri C6 Plus, BD Biosciences).

### LAMP preparation

LAMPs were prepared as described previously (43). Briefly, *M. hyopneumoniae* was cultivated in a mycoplasma medium until the beginning of the stationary growth phase (when a red pH indicator turned orange) and then collected by centrifugation. *M. hyopneumoniae* cells were washed with PBS twice and resuspended in 5 mL of tris-buffered saline (TBS; 50  mM Tris-Cl, pH 8.0, 0.15 M NaCl) containing 1  mM EDTA (TBSE), to which Triton X-114 was added to a final concentration of 2% and incubated at 4°C for 1 hour. The lysate was then incubated at 37°C for 10 minutes for phase separation. After centrifugation, the upper aqueous phase was removed and replaced with the same volume of TBSE. The solution was vortexed and incubated at 4°C for 10 minutes. The phase separation process was repeated twice. The final Triton X-114 phase was resuspended in TBSE to the original volume, and 2.5-fold volumes of ethanol were then added to precipitate the membrane components overnight at −20°C. After centrifugation, the pellet was resuspended in PBS and lysed by sonication. Protein concentrations were examined using the Bradford assay (Thermo Scientific, Waltham, MA, USA). The endotoxin concentration of the heat-inactivated mycoplasma LAMPs was <0.04 endotoxin units/mL, as checked by the Limulus amebocyte lysate assay (Associates of Cape Cod, Falmouth, MA, USA).

### RNA isolation and real-time quantitative RT-PCR

To detect cytokine levels in PAMs, the cells (6.0 × 10^5^ cells/mL) were incubated with PBS or *M. hyopneumoniae* (1.0 × 10^7^ CFU/mL) and followed by the addition of Tu for 6 hours. Total RNA was extracted from the collected cells according to the manufacturer’s instructions for the RNeasy Mini kit (Qiagen Sciences, Hilden, Germany). RNA was reverse-transcribed using the Transcriptor First-Strand cDNA Synthesis Kit (Roche Diagnostics, Indianapolis, USA). Quantitative PCR (Q-PCR) was performed in triplicate using FastStart Universal SYBR Green Master (Rox) (Roche Diagnostics, Indianapolis, USA). All data were acquired with the QuantStudio 5 Real-Time PCR System (Applied Biosystems, Carlsbad, USA). The expression value of each gene was normalized by that of GAPDH. Final values were calculated using the ΔΔCt method. The results were analyzed using QuantStudio design & Analysis software v1.4 (Applied Biosystems). All the primers used in these experiments are summarized in Table 1.

**TABLE 1:** Primers used in this study

Primer name	Sequence (5’−3’)
CD11b-F	AGAAGGAGACACCCAGAGCA
CD11b-R	GTAGGACAATGGGCGTCACT
CD163-F	TTGGGAAAGGAAGTGAGCAG
CD163-R	AATCTCCCATGTGCTTCTCAG
CD204-F	GCCCTTTATCTCCTTGTGTTTG
CD204-R	CACTGTCATTTCCTTTTCCTGC
CD206-F	GCTTGTGGGATGTTTTGAGATG
CD206-R	GCTTGTTTTAGTGGAAGTGCC
CCL22-F	CTCCCTGACACAAGCCTATG
CCL22-R	GATGGATTGGAAGGGTAAGAGG
CCL2-F	ATCTTCAAGACCATCGCGG
CCL2-R	TTCTTGTCCAGGTGGCTTATG
CCL5-F	GATCTCTAGGCTCCGAACTTTG
CCL5-R	CCAGTCATCCTTTCCCAGTG
CD80-F	TTTCAATGTGACAGGCAACC
CD80-R	TGATTAGCAGAAGAGGTTTCTCG
CD86-F	CCGTGCCATTTTACAAAACTCG
CD86-R	AGCTTGTGCGACCCATATAC
NOS2-F	GCAGCTACTGGGTCAAGGAC
NOS2-R	GCTGTTGGTGAACTTCCACTT
TGF-F	CTGGTATCCTTGAAGACACAGG
TGF-R	CGCACCTGAGACATATGGAAG
IL-10-F	TCTGAGAACAGCTGCATCCAC
IL-10-R	CGCCCATCTGGTCCTTCGTT
IL-1β-F	GTGATGCCAACGTGCAGTCT
IL-1β-R	TGGGCCAGCCAGCACTAG
IL-6-F	GGAACGCCTGGAAGAAGATG
IL-6-R	ATCCACTCGTTCTGTGACTG
IL-15-F	ACTGAGGATGGCATTCATGTC
IL-15-R	GCCAGGTTGCTTCTGTTTTAG
TNFα-F	CCCCCAGAAGGAAGAGTTTC
TNFα-R	CGGGCTTATCTGAGGTTTGA

### Cloning and construction of plasmids

The p50ATF6 gene (encoding amino acids 1–373 at the N-terminal of ATF6) was codon-optimized and synthesized by the Genesoul Technology Institute (Harbin, China). Subsequently, this fragment was cloned into the pCAGGS-HA vector.

### Western blot

PTECs and PAMs were harvested at the indicated timepoints after different treatments. An equal number of cells were lysed with the cell lysis buffer (50 mM Tris-HCl, pH 7.4, 150 mM NaCl, 1% Triton X-100, 2 mM EDTA, 0.1% SDS, 5 mM sodium orthovanadate) containing 0.1 mM phenylmethylsulfonyl fluoride (PMSF) and 1 × protease inhibitor cocktail (Roche Molecular Biochemicals) in an ice bath for 30 minutes. The protein concentration was determined using the BCA Protein Assay Kit (Beyotime, Nantong, China). Equal amounts of total cell lysates were separated by SDS-PAGE. The proteins in the gel were transferred onto the nitrocellulose membrane (GE Healthcare Life Science, Piscataway, USA), which were then blocked with 5% skim milk in TBST (20 mM Tris-HCl, pH 7.4, 150 mM NaCl, 0.1% Tween 20) for 1 hour and then incubated for 2 hours with different primary antibodies at room temperature. After washing three times with TBST for 10 minutes each at room temperature, the membrane was incubated with 1: 10,000-diluted DyLight 800-labeled goat anti-mouse or -rabbit IgG (H + L) (1: 10,000, Kirkegaard & Perry Laboratories, Gaithersburg, USA) in TBST for 1 hour at room temperature. The membrane was scanned in an Odyssey Infrared Imaging System (LI-COR Biosciences) after washing with TBST. The fluorescence intensity of each band was measured using Odyssey 2.1 software (LI-COR Biosciences).

Primary antibodies against GRP78 (ab21685), ATF6 (ab37149), CHOP(9C8) (ab11419), phospho-IRE1 (phospho S724) (ab48187), caspase-12 (ab62484), and caspase-9 (ab69514) were purchased from Abcam (Cambridge, MA, USA). Antibodies against total PERK (D11A8) (5683S), p-PERK (16F8) (3179S), total eIF2α (L57A5) (2103S), phospho-eIF2α (D9G8) (3398S), IRE1α (14C10) (3294S), and cleaved caspase-3 (Asp175) (9661S) were purchased from Cell Signaling Technology (Beverly, MA, USA). Anti-caspase-8 (ET1612-70) was purchased from Huaan Biotechnology. Antibody against β-actin (TA-09) was purchased from Zhongshan Goldenbridge-Bio (Beijing, China).

### Statistical analysis

GraphPad Prism software (version 9.0; GraphPad Software Inc.) was used for all statistical analyses. Data obtained from several experiments are reported as the mean  ±  SD. The significance of differences between the two groups was determined with a two-tailed Student’s *t* test. One-way analysis of variances (ANOVA) with Dunnett’s or Tukey’s test was employed for multigroup comparisons. For all analyses, a probability (*P*) value of <0.05 was considered statistically significant.
